# Supplementary material for: A humanized minipig model for the toxicological testing of therapeutic recombinant antibodies
Source: Nat Biomed Eng. 2022 Sep 22;6(11):1248–56. doi: 10.1038/s41551-022-00921-2 (PMC9652145; doi:10.1038/s41551-022-00921-2)
Supplement: Supplementary file 1 — Supplementary figures and tables. [file 41551_2022_921_MOESM1_ESM.pdf]

nature biomedical engineering

Article
https://doi.org/10.1038/s41551-022-00921-2

A humanized minipig model for the toxicological testing of therapeutic recombinant antibodies

In the format provided by the authors and unedited

Contents

Supplementary Fig. 1 l V(D)J sequencing.
Supplementary Fig. 2 l Sequence and structural mapping of the different interactions of PD-L1 epitope amino acid side-chains with atezolizumab paratope residues.

Supplementary Table 1 l List of Ab compounds
Supplementary Table 2 l List of primers.

a

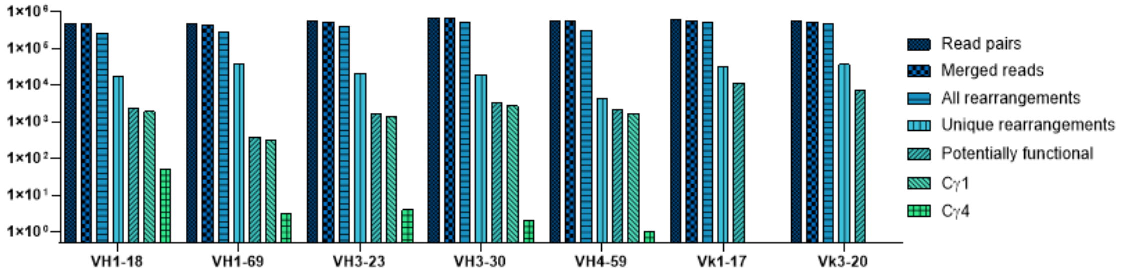

b

```
VH1-69:GTFSSYAISWVRQAPGQGLEWMGGIIPIFGTANYAQKFQGRVTITADKSTSTAYMELSSLRSEDTAVYYCAR  N(D)N        JH                            Cγ
>p1     GTFSSYAISWVRQAPGQGLEWMGWISAYNGNTNYAQKLQGRVTMTTDTSTSTAYMELRSLRSDDTAVYYCA  ANWGY          YFDYWGQGTLVTVSS(4)ASTKGPSVFPLAPSSKSTSGGTAAL
>p45    GTFSSYAISWVRQAPGQGLEWMGWISAYNGNTNYAQKLQGRVTMTTDTSTSTAYMELRSLRSDDTAVYYCA  ANWGY          YFDYWGQGTLVTVSS(4)ASTKGPSVFPLAPCSRSTSESTAAL
>p2     GTFSSYAISWVRPAPGQGLEWMGWISAYNGNTNYAQKLQGRVTMTTDTSTSTAYMELRSLRSDDTAVYYCA  ANWGY          YFDYWGQGTLVTVSS(4)ASTKGPSVFPLAPSSKSTSGGTAAL
>p3     GTFSSYAISWVRQAPGQGLEWMGWISAYNGNTNYAQKLQGRVTMTTDTSTSTAYMELRSLRSDDTAVYYCA  NWG       YYYYYMDVWGKGTTVTVSS(6)ASTKGPSVFPLAPSSKSTSGGTAAL
>p6     GTFSSYAISWVRQAPGQGLEWMGWISAYNGNTNYAQKLQGRVTMTTDTSTSTAYMELRSLRSDDTAVYYCA  KD             YFDYWGQGTLVTVSS(4)ASTKGPSVFPLAPSSKSTSGGTAAL
>p8     GTFSSYAISWVRQAPGQGLEWMGWISAYNGNTNYAQKLQGRVTMTTDTSTSTAYMELRSLRSDDTAVYYCA  KDY              DYWGQGTLVTVSS(4)ASTKGPSVFPLAPSSKSTSGGTAAL
>p9     GTFSSYAISWVRQAPGQGLEWMGWISAYNGNTNYAQKLQGRVTMTTDTSTSTAYMELRSLRSDDTAVYYCA  KN                YWGQGTLVTVSS(4)ASTKGPSVFPLAPSSKSTSGGTAAL
>p10    GTFSSYAISWVRQAPGQGLEWMGWISAYNGNTNYAQKLQGRVTMTTDTSTSTAYMELRSLRSDDTAVYYCA  KDLY           YFDYWGQGTLVTVSS(4)ASTKGPSVFPLAPSSKSTSGGTAAL
>p11    GTFSSYAISWVRQAPGQGLEWMGWISAYNGNTNYAQKLQGRVTMTTDTSTSTAYMELRSLRSDDTAVYYCA  NC         YYYYMDVWGKGTTVTVSS(6)ASTKGPSVFPLAPSSKSTSGGTAAL
>p12    GTFSSYAISWVRQAPGQGLEWMGWISAYNGNTNYAQKLQGRVTMTTDTSTSTAYMELRSLRSDDTAVYYCA  QRTG      YYYYYMDVWGKGTTVTVSS(6)ASTKGPSVFPLAPSSKSTSGGTAAL
>p13    GTFSSYAISWVRQAPGQGLEWMGWISAYNGNTNYAQKLQGRVTMTTDTSTSTAYMELRSLRSDDTAVYYCA  KLD            YFDYWGQGTLVTVSS(4)ASTKGPSVFPLAPSSKSTSGGTAAL
>p14    GTFSSYAISWVRQAPGQGLEWMGWISAYNGNTNYAQKLQGRVTMTTDTSTSTAYMELRSLRSDDTAVYYCA  KDRGF             DYWGQGTLVTVSS(4)ASTKGPSVFPLAPSSKSTSGGTAAL
>p22    GTFSSYAISWVRQAPGQGLEWMGWISAYNGNTNYAQKLQGRVTMTTDTSTSTAYMELRSLRSDDTAVYYCA  KDRGF             DYWGQGTLVTVSS(4)ASTKGPSVFPLAPSSNSTSGGTAAL
>p15    GTFSSYAISWVRQAPGQGLEWMGWISAYNGNTNYAQKLQGRVTMTTDTSTSTAYMELRSLRSDDTAVYYCA  ANWGY          YFDYWGRGTLVTVSS(4)ASTKGPSVFPLAPSSKSTSGGTAAL
>p16    GTFSSYAISWVRQAPGQGLEWMGWISAYNGNTNYAQKLQGRVTMTTDTSTSTAYMELRSLRSDDTAVYYCA  KGPVCGWFDP          WGQGTLVTVSS(4)ASTKGPSVFPLAPSSKSTSGGTAAL
>p17    GTFSSYAISWVRQAPGQGLEWMGWISAYNGNTNYAQKLQGRVTMTTDTSTSTAYMELRSLRSDDTAVYYCA  KDLGS              YWGQGTLVTVSS(4)ASTKGPSVFPLAPSSKSTSGGTAAL
>p19    GTFSSYAISWVRQAPGQGLEWMGWISAYNGNTNYAQKLQGRVTMTTDTSTSTAYMELRSLRSDDTAVYYCA  KD                DYWGQGTLVTVSS(4)ASTKGPSVFPLAPSSKSTSGGTAAL
>p21    GTFSSYAISWVRQAPGQGLEWMGWISAYNGNTNYAQKLQGRVTMTTDTSTSTAYMELRSLRSDDTAVYYC   SANWGY         YFDYWGQGTLVTVSS(4)ASTKGPSVFPLAPSSKSTSGGTAAL
>p23    GTFSSYAISWVRQAPGQGLEWMGWISAYNGNTNYAQKLQGRVTMTTDTSTSTAYMELRSLRSDDTAVYYCA  K            DAFDIWGQGTMVTVSS(3)ASTKGPSVFPLAPSSKSTSGGTAAL
>p24    GTFSSYAISWVRQAPGQGLEWMGWISAYNGNTNYAQKLQGRVTMTTDTSTSTAYMELRSLRSDDTAVYYCA  ANWG      YYYYYMDVWGKGTTVTVSS(6)ASTKGPSVFPLAPSSKSTSGGTAAL
>p29    GTFSSYAISWVRQAPGQGLEWMGWISAYNGNTNYAQKLQGRVTMTTDTSTSTAYMELRSLRSDDTAVYYCA  ANWGY          YLDYWGQGTLVTVSS(4)ASTKGPSVFPLAPSSKSTSGGTAAL
>p34    GTFSSYAISWVRQAPGQGLEWMGWISAYNGNTNYAQKLQGRVTMTTDTSTSTAYMELRSLRSDDTAVYYCA  KDL      YYYYYYMDVWGKGTTVTVSS(6)ASTKGPSVFPLAPSSKSTSGGTAAL
>p183   GTFSSYAISWVRQAPGQGLEWMGWISAYNGNTNYAQKLQGRVTMTTDTSTSTAYMELRSLRSDDTAVYYCA  KDL      YYYYYYMDVWGKGTTVTVSS(6)ASTKGPSVFPLAPCSRSTSESTAAL
>p35    GTFSSYAISWVRQAPGQGLEWMGWISAYNGNTNYAQKLQGRVTMTTDTSTSTAYMELRSLRSDDTAVYYCA  KDKVSNY   YYYYYMDVWGKGTTVTVSS(6)ASTKGPSVFPLAPSSKSTSGGTAAL
>p41    GTFSSYAISWVRQAPGQGLEWMGWISAYNGNTNYAQKLQGRVTMTTDTSTSTAYMELRSLRSDDTAVYYCA  KSPAY     YYYYYMDVWGKGTTVTVSS(6)ASTKGPSVFPLAPSSKSTSGGTAAL
>p42    GTFSSYAISWVRQAPGQGLEWMGWISAYNGNTNYAQKLQGRVTMTTDTSTSTAYMELRSLRSDDTAVYYCA  KERGHH    YYYYYMDVWGKGTTVTVSS(6)ASTKGPSVFPLAPSSKSTSGGTAAL
>p180   GTFSSYAISWVRQAPGQGLEWMGWISAYNGNTNYAQKLQGRVTMTTDTSTSTAYMELRSLRSDDTAVYYCA  KD             YFDYWGQGTLVTVSS(4)ASIKGPSVFPLAPSSKSTSGGTAAL
>p181   GTFSSYAISWVRQAPGQGLEWMGWISAYNGNTNYAQKLQGRVTMTTDTSTSTAYMELRSLRSDDTAVYYCA  KD              YDYWGQGTLVTVSS(4)ASTKSPSVFPLAPSSKSTSGGTAAL
>p182   GTFSSYAISWVRQAPGQGLEWMGWISAYNGNTNYAQKLQGRVTMTTDTSTSTAYMELRSLRSDDTAVYYCA  KDYD           YFDYWGQGTLVTVSS(4)ASTKGPSVFPLAPSSKSTSGGTAAL
>p184   GTFSSYAISWVRQAPGQGLEWMGWISAYNGNTNYAQKLQGRVTMTTDTSTSTAYMELRSLRSDDTAVYYCA  KDLG           YFDYWGQGTLVTVSS(4)ASTKGPSVFPLAPSSKSTSGGTAAL
>p267   GTFSSYAISWVRQAPGQGLEWMGWISAYNGNTNYAQKLQGRVTMTTDTSTSTAYMELRSLRSDDTAVYYCA  ANLGY          YFDYWGQGTLVTVSS(4)ASTKGPSVFPLAPCSRSTSESTAAL
```

```
VH4-59: KGLEWIGYIYYSGSTNYNPSLKSRVTISVDTSKNQFSLKLSSVTAADTAVYYCAR N(D)N      JH                    Cγ
>p1     KGLEWIGYIYYSGSTNYNPSLKSRVTISVDTSKNQFSLKLSSVTAADTAVYYCAR DLGI       DYWGQGTLVTVSS(4)ASTKGPSVFPLAPSSKSTSGGTAAL
>p2     KGLEWIGYIYYSGSTNYNPSLKSRVTISVDTSKNQFSLKLSSVTAADTAVYYCAR EG    YYYMDVWGKGTTVTVSS(6)ASTKGPSVFPLAPSSKSTSGGTAAL
>s63    KGLEWIGYIYYSGSTNYNPSLKSRVTISVDTSKNQFSLKLSSVTAADTAVYYCAR DLGIG       YWGQGTLVTVSS(4)ASTKGPSVFPLAPSSKSTSGGTAAL
>p79    KGLEWIGYIYYSGSTNYNPSLKSRVTISVDTSKNQFSLKLSSVTAADTAVYYCA  SN           WGQGTLVTVSS(4)ASTKGPSVFPLAPSSKSTSGGTAAL
>p80    KGLEWIGYIYYSGSTNYNPSLKSRVTISVDTSKNQFSLKLSSVTAADTAVYYCAR DRGI       DYWGQGTLVTVSS(4)ASTKGPSVFPLAPSSKSTSGGTAAL
>p81    KGLEWIGYIYYSGSTNYNPSLKSRVTISVDTSKNQFSLKLSSVTAADTAVYYCAR DQGI       DYWGQGTLVTVSS(4)ASTKGPSVFPLAPSSKSTSGGTAAL
>p82    KGLEWIGYIYYSGSTNYNPSLKSRVTISVDTSKNQFSLKLSSVTAADTAVYYCAR DLVI       DYWGQGTLVTVSS(4)ASTKGPSVFPLAPSSKSTSGGTAAL
>p182   KGLEWIGYIYYSGSTNYNPSLKSRVTISVDTSKNQFSLKLSSVTAADTAVYYCAR DLVI       DYWGQGTLVTVSS(4)ASTKGPSVFPLAPSSKSTSGGTAAL
```

```
VH3-30:  ADSVKGRFTISRDNSKNTLYLQMNSLRAEDTAVYYCAK N(D)N            JH                    Cγ
>p1      ADSVKGRFTISRDNSKNTLYLQMNSLRAEDTAVYYCPK EGR                 GQGTLVTVSS(4)ASTKGPSVFPLAPSSKSTSGGTAAL
>p2      ADSVKGRFTISRDNSKNTLYLQMNSLRAEDTAVYYCAK               DAFDIWGQGTMVTVSS(3)ASTKGPSVFPLAPSSKSTSGGTAAL
>p3      ADSVKGRFTISRDNSKNTLYLQMNSLRAEDTAVYYCAK DPY            YFDYWGQGTLVTVSS(4)ASTKGPSVFPLAPSSKSTSGGTAAL
>p4      ADSVKGRFTISRDNSKNTLYLQMNSLRAEDTAVYYCA  NMVRE           FDYWGQGTLVTVSS(4)ASTKGPSVFPLAPSSKSTSGGTAAL
>p5      ADSVKGRFTISRDNSKNTLYLQMNSLRAEDTAVYYCAK ANWG            FDYWGQGTLVTVSS(4)ASTKGPSVFPLAPSSKSTSGGTAAL
>p6      ADSVKGRFTISRDNSKNTLYLQMNSLRAEDTAVYYCAK DQLANWGS        FDIWGQGTMVTVSS(3)ASTKGPSVFPLAPSSKSTSGGTAAL
>p8      ADSVKGRFTISRDNSKNTLYLQMNSLRAEDTAVYYCAK EGR                 GQGTLVTVSS(4)ASTKGPSVFPLAPSSKSTSGGTAAL
>p15     ADSVKGRFTISRDNSKNTLYLQMNSLRAEDTAVYYCAK DQLANWG         FDYWGQGTLVTVSS(4)ASTKGPSVFPLAPSSKSTSGGTAAL
>p17     ADSVKGRFTISRDNSKNTLYLQMNSLRAEDTAVYYCAK ANWGS           FDIWGQGTMVTVSS(3)ASTKGPSVFPLAPSSKSTSGGTAAL
>p18     ADSVKGRFTISRDNSKNTLYLQMNSLRAEDTAVYYCAK DTNWG           FDYWGQGTLVTVSS(4)ASTKGPSVFPLAPSSKSTSGGTAAL
>p23     ADSVKGRFTISRDNSKNTLYLQMNSLRAEDTAVYYCAK AHWG            FDYWGQGTLVTVSS(4)ASTKGPSVFPLAPSSKSTSGGTAAL
>p24     ADSVKGRFTISRDNSKNTLYLQMNSLRAEDTAVYYCAK DTNWGS          FDIWGQGTMVTVSS(3)ASTKGPSVFPLAPSSKSTSGGTAAL
>p28     ADSVKGRFTISRDNSKNTLYLQMNSLRAEDTAVYYCAK DTNWG                QGTLVTVSS(4)ASTKGPSVFPLAPSSKSTSGGTAAL
>p34     ADSVKGRFTISRDNSKNTLYLQMNSLRAEDTAVYYCAK DQFANWGS        FDIWGQGTMVTVSS(3)ASTKGPSVFPLAPSSKSTSGGTAAL
>p39     ADSVKGRFTISRDNSKNTLYLQMNSLRAEDTAVYYCAK DQLANWGS       AFDIWGQGTMVTVSS(3)ASTKGPSVFPLAPSSKSTSGGTAAL
>p1763   ADSVKGRFTISRDNSKNTVYLQMNSLRREDTAVYYCA  RVEGRQGNSNCPL DYWGQGTLVTVSS(4)ASTKGPSVFPLAPCSRSTSESTAAL
>p3138   ADSVKGRFTISRDDSKNTLYLQMNSLRVEDTAVYFCA  REGDDRAYYSA WFDPWGQGTLVTVSS(4)ASTKGPSVFPLAPCSRSTSESTAAL
```

VH3-23:	ADSVKGRFTISRDNSKNTLYLQMNSLRAEDTAVYYCAK	N(D)N	JH	Cγ	
>p1	ADSVKGRFTISRDNSKNTLYLQMNSLRAEDTAVYYCAK	DYYGSGSY	DAFDIWGQGTMVTVSS(3)	ASTKGPSVFPLAPSSKSTSGGTAAL	D3-9
>p2	ADSVKGRFTISRDNSKNTLYLQINSLRAEDTAVYYCAK	DRG	FDYWGQGTLVTVSS(4)	ASTKGPSVFPLAPSSKSTSGGTAAL	
>p4	ADSVKGRFTISRDNSKNTLYLQMNSLRAEDTAVYYCAK	LY	YFDYWGQGTLVTVSS(4)	ASTKGPSVFPLAPSSKSTSGGTAAL	
>p5	ADSVKGRFTISRDNSKNTLYLQMNSLRAEDTAVYYCA	ITGV	DYWGQGTLVTVSS(4)	ASTKGPSVFPLAPSSKSTSGGTAAL	
>p6	ADSVKGRFTISRDNSKNTLYLQMNSLRAEDTAVYYCAK	APP	FDIWGQGTMVTVSS(3)	ASTKGPSVFPLAPSSKSTSGGTAAL	
>p7	ADSVKGRFTISRDNSKNTLYLQMNSLRAEDTAVYYCA	N	YWGQGTLVTVSS(4)	ASTKGPSVFPLAPSSKSTSGGTAAL	
>p8	ADSVKGRFTISRDNSKNTLYLQMNSLRAEDTAVYYCAK	D	YWGQGTLVTVSS(4)	ASTKGPSVFPLAPSSKSTSGGTAAL	
>p9	ADSVKGRFTISRDNSKNTLYLQMNSLRAEDTAVYYCAK	DGTC	YYYYMDVWGKGTTVTVSS(6)	ASTKGPSVFPLAPSSKSTSGGTAAL	
>p10	ADSVKGRFTISRDNSKNTLYLQMNSLRAEDTAVYYCAK	ES	YYYYMDVWGKGTTVTVSS(6)	ASTKGPSVFPLAPSSKSTSGGTAAL	
>p11	ADSVKGRFTISRDNSKNTLYLQMNSLRAEDTAV	AN	YYYYMDVWGKGTTVTVSS(6)	ASTKGPSVFPLAPSSKSTSGGTAAL	
>p12	ADSVKGRFTISRDNSKNTLYLQMNSLRAEDTAVYYCAK	DYYGSGSYYN	YYYYMDVWGKGTTVTVSS(6)	ASTKGPSVFPLAPSSKSTSGGTAAL	D3-9
>p14	ADSVKGRFTISRDNSKNTLYLQMNSLRAEDTAVYYCAK	SLDP	WGQGTLVTVSS(4)	ASTKGPSVFPLAPSSKSTSGGTAAL	
>p15	ADSVKGRFTISRDNSKNTLYLQMNSLRAEDTAVYYCAK	DFG	DAFDIWGQGTMVTVSS(3)	ASTKGPSVFPLAPSSKSTSGGTAAL	
>p17	ADSVKGRFTISRDNSKNTLYLQMNSLRAEDTAVYYCAK	D	FDYWGQGTLVTVSS(4)	ASTKGPSVFPLAPSSKSTSGGTAAL	
>p18	ADSVKGRFTISRDNSKNTLYLQMNSLRAEDTAVYYCAK	S	YWYFDLWGRGTLVTVSS(2)	ASTKGPSVFPLAPSSKSTSGGTAAL	
>p19	ADSVKGRFTISRDNSKNTLYLQMNSLRAEDTAVYYCAK	T	DAFDIWGQGTMVTVSS(3)	ASTKGPSVFPLAPSSKSTSGGTAAL	
>p20	ADSVKGRFTISRDNSKNTLYLQMNSLRAEDTAVYYCAK	VYYGSGSY	DAFDIWGQGTMVTVSS(3)	ASTKGPSVFPLAPSSKSTSGGTAAL	D3-9
>p22	ADSVKGRFTISRDNSKNTLYLQMNSLRAEDTAVYYCAK	DLD	YFDYWGQGTLVTVSS(4)	ASTKGPSVFPLAPSSKSTSGGTAAL	
>p24	ADSVKGRFTISRDNSKNTLYLQMNSLRAEDTAVYYCAK	ELT	DAFDIWGQGTMVTVSS(3)	ASTKGPSVFPLAPSSKSTSGGTAAL	
>p25	ADSVKGRFTISRDNSKNTLYLQMNSLRAEDTAVYYCAK	DG	YFDYWGQGTLVTVSS(4)	ASTKGPSVFPLAPSSKSTSGGTAAL	
>p26	ADSVKGRFTISRDNSKNTLYLQMNSLRAEDTAVYYCAK	EY	YFDYWGQGTLVTVSS(4)	ASTKGPSVFPLAPSSKSTSGGTAAL	
>p27	ADSVKGRFTISRDNSKNTLYLQMNSLRAEDTAV	LHYDH	AFDIWGQGTMVTVSS(3)	ASTKGPSVFPLAPSSKSTSGGTAAL	
>p28	ADSVKGRFTISRDNSKNTLYLQMNSLRAEDTAVYYCA	SWGY	YYYYYMDVWGKGTTVTVSS(6)	ASTKGPSVFPLAPSSKSTSGGTAAL	
>p29	ADSVKGRFTISRDNSKNTLYLQMNSLRAEDTAVYYCAK		DAFDIWGQGTMVTVSS(3)	ASTKGPSVFPLAPSSKSTSGGTAAL	
>p30	ADSVKGRFTISRDNSKNTLYLQMNSLRAEDTAVYYCA	NWG	YYYYYMDVWGKGTTVTVSS(6)	ASTKGPSVFPLAPSSKSTSGGTAAL	
>p31	ADSVKGRFTISRDNSKNTLFLQMNSLRAEDTAVYYCAK	DRGF	DYWGQGTLVTVSS(4)	ASTKGPSVFPLAPSSKSTSGGTAAL	
>p32	ADSVKGRFTISRDNSKNTLYLQMNSLRAEDTAVYYCAK	DPRDY	YYYYYMDVWGKGTTVTVSS(6)	ASTKGPSVFPLAPSSKSTSGGTAAL	
>p33	ADSVKGRFTISRDNSKNTLYLQMNSLRAEDTAVYYCA	IITGD	YYYYYMDVWGKGTTVTVSS(6)	ASTKGPSVFPLAPSSKSTSGGTAAL	
>p34	ADSVKGRFTISRDNSKNTLYLQMNSLRAEDTAVYYCAK	WGS	FDYWGQGTLVTVSS(4)	ASTKGPSVFPLAPSSKSTSGGTAAL	
>p36	ADSVKGRFTISRDNSKNTLYLQMNSLRAEDTAVYYCAK	DKVSNY	YYYYYMDVWGKGTTVTVSS(6)	ASTKGPSVFPLAPSSKSTSGGTAAL	
>p37	ADSVKGRFTISRDNSKNTLYLQMNSLRAEDTAVYYCAK	D	FDIWGQGTMVTVSS(3)	ASTKGPSVFPLAPSSKSTSGGTAAL	
>p38	ADSVKGRFTISRDNSKNTLYLQMNSLRAEDTAVYYCAK	VLAGPPD	YYYYYMDVWGKGTTVTVSS(6)	ASTKGPSVFPLAPSSKSTSGGTAAL	
>p39	ADSVKGRFTISRDNSKNTLYLQMNSLRAEDTAVYYCAK	GGGSGSY	DAFDIWGQGTMVTVSS(3)	ASTKGPSVFPLAPSSKSTSGGTAAL	D3-9
>p41	ADSVKGRFTISRDNSKNTLYLQMNSLRAEDTAVYYCAK	DLGI	YYYYYMDVWGKGTTVTVSS(6)	ASTKGPSVFPLAPSSKSTSGGTAAL	
>p42	ADSVKGRFTISRDNSKNTLYLQMNSLRAEDTAVYYCAK	DYGSGSYY	NWFDPWGQGTLVTVSS(5)	ASTKGPSVFPLAPSSKSTSGGTAAL	D3-9
>p43	ADSVKGRFTISRDNSKNTLYLQMNSLRAEDTAVYYCAK	DLTGV	DYWGQGTLVTVSS(4)	ASTKGPSVFPLAPSSKSTSGGTAAL	
>p44	ADSVKGRFTISRDNSKNTLYLQMNSLRAEDTAVYYCAK	TGDF	FDYWGQGTLVTVSS(4)	ASTKGPSVFPLAPSSKSTSGGTAAL	
>p45	ADSVKGRFTISRDNSKNTLYLQMNSLRADDTAVYYCAK	DKVSNY	YYYYYMDVWGKGTTVTVSS(6)	ASTKGPSVFPLAPSSKSTSGGTAAL	
>p46	ADSVKGRFTISRDNSKNTLYLQMNSLRAEDTAVYYC	RLTGD	YYYYYMDVWGKGTTVTVSS(6)	ASTKGPSVFPLAPSSKSTSGGTAAL	
>p47	ADSVKGRFTISRDNSKNTLYLQMNSLRAEDTAVYYCAK	D	YFDYWGQGTLVTVSS(4)	ASTKGPSVFPLAPSSKSTSGGTAAL	
>p48	ADSVKGRFTISRDNSKNTLYLQMNSLRAEDTAVYYCAK	DY	DYWGQGTLVTVSS(4)	ASTKGPSVFPLAPSSKSTSGGTAAL	
>p50	ADSVKGRFTISRDNSKNTLYLQMNSLRAEDTAVYYCAK	D	DYWGQGTLVTVSS(4)	ASTKGPSVFPLAPSSKSTSGGTAAL	
>p596	ADSVKGRFTISRDNSKNTLYLQMNSLRVEDTAVYYCAK	DDRPWLKWFGETS	MDVWGQGTTVTVSS(6)	ASTKGPSVFPLAPCSRSTSESTAAL	
>p1528	ADSVKGRFTISRDNSKNMLYLQMNSLRAGDTAVYYCA	RQEYSSSYKWFDP	WGQGTLVTVSS(6)	ASTKGPSVFPLAPCSRSTSESTAAL	
>p1565	ADSVKGRFTISRDNAKNTLFLQMNSLRAEDTAVYYCA	RFSSESPDYF	YGLDVWGQGTMVTVSS(6)	ASTKGPSVFPLAPCSRSTSESTAAL	

VH1-18:	GISWVRQAPGQGLEWMGWISAYNGNTNYAQKLQGRVTMTTDTSTSTAYMELRSLRSDDTAVYYCAR	N(D)N	JH	Cγ
>p1	GISWVRQAPGQGLEWMGWISAYNGNTNYAQKLQGRVTMTTDTSTSTAYMELRSLRSDDTAVYYCAR	V	YFDLWGRGTLVTVSS(2)	ASTKGPSVFPLAPSSKSTSGGTAAL
>p8	GISWVRQAPGQGLEWMGWISAYNGNTNYAQKLQGRVTMTTDTSTSTAYMELRSLRSDDTAVYYCAR	V	YFDLWGRGTLVTVSS(2)	ASTKGPSVFPLAPCSRSTSESTAAL
>p2	GISWVRQAPGQGLEWMGWISAYNGNTNYAQKLQGRVTMTTDTSTSTAYMELRSLRSDDTAVYYCAR	D	DYWGQGTLVTVSS(4)	ASTKGPSVFPLAPSSKSTSGGTAAL
>p10	GISWVRQAPGQGLEWMGWISAYNGNTNYAQKLQGRVTMTTDTSTSTAYMELRSLRSDDTAVYYCAR	D	DYWGQGTLVTVSS(4)	ASTKGPSVFPLAPCSRSTSESTAAL
>p15	GISWVRQAPGQGLEWMGWISAYNGNTNYAQKLQGRVTMTTDTSTSTAYMELRSLRSDDTAVYYCAR	D	DYWGRGTLVTVSS(4)	ASTKGPSVFPLAPSSKSTSGGTAAL
>p3	GISWVRQAPGQGLEWMGWISAYNGNTNYAQKLQGRVTMTTDTSTSTAYMELRSLRSDDTAVYYCAR		AFDIWGQGTMVTVSS(3)	ASTKGPSVFPLAPSSKSTSGGTAAL
>p14	GISWVRQAPGQGLEWMGWISAYNGNTNYAQKLQGRVTMTTDTSTSTAYMELRSLRSDDTAVYYCAR		AFDIWGQGTMVTVSS(3)	ASTKGPSVFPLAPCSRSTSESTAAL
>p4	GISWVRQAPGQGLEWMGWISAYNGNTNYAQKLQGRVTMTTDTSTSTAYMELRSLRSDDTAVYYCA	S	DYWGQGTLVTVSS(4)	ASTKGPSVFPLAPSSKSTSGGTAAL
>p13	GISWVRQAPGQGLEWMGWISAYNGNTNYAQKLQGRVTMTTDTSTSTAYMELRSLRSDDTAVYYCA	S	DYWGQGTLVTVSS(4)	ASTKGPSVFPLAPCSRSTSESTAAL
>p26	GISWVRQAPGQGLEWMGWISAYNGNTNYAQKLQGRVTMTTDTSTSTAYMELRSLRSDDTAVYYCAR	N	WGQGTLVTVSS(4)	ASTKGPSVFPLAPSSKSTSGGTAAL
>p30	GISWVRQAPGQGLEWMGWISAYNGNTNYAQKLQGRVTMTTDTSTSTAYMELRSLRSDDTAVYYCAR	DD	YYYYMDVWGKGTTVTVSS(6)	ASTKGPSVFPLAPSSKSTSGGTAAL
>p41	GISWVRQAPGQGLEWMGWISAYNGNTNYAQKLQGRVTMTTDTSTSTAYMELRSLRSDDTAVYYCAR	AGMGV	WGKGTTVTVSS(6)	ASTKGPSVFPLAPSSKSTSGGTAAL
>p52	GISWVRQAPGQGLEWMGWISAYNGNTNYAQKLQGRVTMTTDTSTSTAYMELRSLRSDDTAVYYCAR	DHWGL	YFDLWGRGTLVTVSS(2)	ASTKGPSVFPLAPSSKGTSGGTAAL
>p113	GISWVRQAPGQGLEWMGWISAYNGNTNYAQKLQGRVTMTTDTSTSTAYMELRSLRSDDTAVYYCAR	DHWGL	YFDLWGRGTLVTVSS(2)	ASTKSPSVFPLAPCSRSTSESTAAL
>p60	GISWVRQAPGQGLEWMGWISAYNGNTNYAQKLQGRVTMTTDTSTSTAYMELRSLRSDDTAVYYCAR	GY	YFDYWGQGTLVTVSS(4)	ASTKGPSVFPLAPCSRSTSGGTAAL
>p83	GISWVRQAPGQGLEWMGWISAYNGNTNYAQKLQGRVTMTTDTSTSTAYMELRSLRSDDTAVYYCAR	GY	YFDYWGRGTLVTVSS(4)	ASTKGPSVFPLAPCSRSTSESTAAL
>p67	GISWVRQAPGQGLEWMGWISAYNGNTNYAQKLQGRVTMTTDTSTSTAYMELRSLRSDDTAVYYCA	SY	YFDYWGQGTLVTVSS(4)	ASTKGPSVFPLAPSSKSTAGGTAAL
>p73	GISWVRQAPGQGLEWMGWISAYNGNTNYAQKLQGRVTMTTDTSTSTAYMELRSLRSDDTAVYYCA	GDHWGL	YFDLWGRGTLVTVSS(2)	ASTKGPSVFPLAPSSKSTSGGTAAL
>p96	GISWVRQAPGQGLEWMGWISAYNGNTNYAQKLQGRVTMTTDTSTSTAYMELRSLRSDDTAVYYCA	SDHWGL	YFDLWGRGTLVTVSS(2)	ASTKGPSVFPLAPSSKSTSGGTAAL

```
VK3-20:GTLSLSPGERATLSCRASQSVSSSYLAWYQQKPGQAPRLLIYGASSRATGIPDRFSGSGSGTDFTLTISRLEPEDFAVYYCQQYGSSP  N          Jκ
>p1    GTLSLSPGERATLSCRASQSVSSSYLAWYQQKPGQAPRLLIYGASSRATGIPDRFSGSGSGTDFTLTISRLEPEDFAVYYCQQYGSSP  PNWVTNWG  TFGPGTKVDIK(3)
>p2    GTLSLSPGERATLSCRASQSVSSSYLAWYQQKPGQAPRLLIYGASSRATGIPDRFSGSGSGTDFTLTISRLEPEDFAVYYCQQYGSSP            YTFGQGTKLEIK(2)
>p3    GTLSLSPGERATLSCRASQSVSSSYLAWYQQKPGQAPRLLIYGASSRATGIPDRFSGSGSGTDFTLTISRLEPEDFAVYYCQQYGRS             RSFGGGTKVEIK(4)
>p4    GTLSLSPGERATLSCRASQSVSSSYLAWYQQKPGQAPRLLIYGASSRATGIPDRFSGSGSGTDFTLTISRLEPEDFAVYYCQQYGSSP            LTFGGGTKVEIK(4)
>p5    GTLSLSPGERATLSCRASQSVSSSYLAWYQQKPGQAPRLLIYGASSRATGIPDRFSGSGSGTDFTLTISRLEPEDFAVYYCQQYGSS   DYNHTP   RTFGQGTKVEIK(1)
>p6    GTLSLSPGERATLSCRASQSVSSSYLAWYQQKPGQAPRLLIYGASSRATGIPDRFSGSGSGTDFTLTISRLEPEDFAVYYCQQYGSS   RECE     WTFGQGTKVEIK(1)
>p7    GTLSLSPGERATLSCRASQSVSSSYLAWYQQKPGQAPRLLIYGASSRATGIPDRFSGSGSGTDFTLTISRLEPEDFAVYYCQQYGSS   PM       YTFGQGTKLEIK(2)
>p8    GTLSLSPGERATLSCRASQSVSSSYLAWYQQKPGQAPRLLIYGASSRATGIPDRFSGSGSGTDFTLTISRLEPEDFAVYYCQQYGSS   VVYKK    YTFGQGTKLEIK(2)
>p9    GTLSLSPGERATLSCRASQSVSSSYLAWYQQKPGQAPRLLIYGASSRATGIPDRFSGSGSGTDFTLTISRLEPEDFAVYYCQQYGSS               RGGTKVEIK(4)
>p11   GTLSLSPGERATLSCRASQSVSSSYLAWYQQKPGQAPRLLIYGASSRATGIPDRFSGSGSGTDFTLTISRLEPEDFAVYYCQQYGSSQNATTVQPLWYTFGQGTKLEIK(2)
>p12   GTLSLSPGERATLSCRASQSVSSSYLAWYQQKPGQAPRLLIYGASSRATGIPDRFSGSGSGTDFTLTISRLEPEDFAVYYCQQYGSSP  PM       YTFGQGTKLEIK(2)
>p14   GTLSLSPGERATLSCRASQSVSSSYLAWYQQKPGQAPRLLIYGASSRATGIPDRFSGSGSGTDFTLTISRLEPEDFAVYYCQQYGSSP            FTFGPGTKVDIK(3)
>p15   GTLSLSPGERATLSCRASQSVSSSYLAWYQQKPGQAPRLLIYGASSRATGIPDRFSGSGSGTDFTLTISRLEPEDFAVYYCQQYGS    LT       WTFGQGTKVEIK(1)
>p16   GTLSLSPGERATLSCRASQSVSSSYLAWYQQKPGQAPRLLIYGASSRATGIPDRFSGSGSGTDFTLTISRLEPEDFAVYYCQQYGSSP  PTVM     IIIGPGTKVDIK(3)
>p17   GTLSLSPGERATLSCRASQSVSSSYLAWYQQKPGQAPRLLIYGASSRATGIPDRFSGSGSGTDFTLTISRLEPEDFAVYYCQQYGSSP             TFGQGTKLEIK(2)
>p18   GTLSLSPGERATLSCRASQSVSSSYLAWYQQKPGQAPRLLIYGASSRATGIPDRFSGSGSGTDFTLTISRLEPEDFAVYYCQQYGSSP  PCARMHV   SFGPGTKVDIK(3)
>p19   GTLSLSPGERATLSCRASQSVSSSYLAWYQQKPGQAPRLLIYGASSRATGIPDRFSGSGSGTDFTLTISRLEPEDFAVYYCQQYGSSP            PTFGQGTKVEIK(1)
>p20   GTLSLSPGERATLSCRASQSVSSSYLAWYQQKPGQAPRLLIYGASSRATGIPDRFSGSGSGTDFTLTISRLEPEDFAVYYCQQYDSS   YRLTD    YTFGQGTKLEIK(2)
>p21   GTLSLSPGERATLSCRASQSVSSSYLAWYQQKPGQAPRLLIYGASSRATGIPDRFSGSGSGTDFTLTISRLEPEDFAVYYCQQYGSS   LKG      RTFGQGTKVEIK(1)
>p22   GTLSLSPGERATLSCRASQSVSSSYLAWYQQKPGQAPRLLIYGASSRATGIPDRFSGSGSGTDFTLTISRLEPEDFAVYYCQQYGSSP            HTFGQGTKLEIK(2)
>p23   GTLSLSPGERATLSCRASQSVSSSYLAWYQQKPGQAPRLLIYGASSRATGIPDRFSGSGSGTDFTLTISRLEPEDFAVYYCQQYGSS   GPDKPPM  YTFGQGTKLEIK(2)
>p24   GTLSLSPGERATLSCRASQSVSSSYLAWYQQKPGQAPRLLIYGASSRATGIPDRFSGSGSGTDFTLTISRLEPEDFAVYYCQQYGSS            REFGQGTKVEIK(1)
>p25   GTLSLSPGERATLSCRASQSVSSSYLAWYQQKPGQAPRLLGYGASSRATGIPDRFSGSGSGTDFTLTISRLEPEDFAVYYCQQYGSSP  TKS      YSFGPGTKVDIK(3)
>p26   GTLSLSPGERATLSCRASQSVSSSYLAWYQQKPGQAPRLLIYGASSRATGIPDRFSGSGSGTDFTLTISRLEPEDFAVYYCQQYGSSP  HAPPRT   WTFGQGTKVEIK(1)
>p27   GTLSLSPGERATLSCRASQSVSSSYLDWYQQKPGQAPRLLIYGASSRATGIPDRFSGSGSGADFTLTISRLEPEDFAVYYCQQYGSS   L        YTFGQGTKLEIK(2)
>p28   GTLSLSPGERATLSCRASQSVSSSYLAWYQQKPGQAPRLLIYGASSRATGIPDRFSGSGSGTDFTLTISRLEPEDFAVYYCQQYGSS   SP         FGPGTKVDIK(3)
>p29   GTLSLSPGERATLSCRASQSVSSSYLAWYQQKPGQAPRLLIYGASSRATGIPDRFSGSGSGTDFTLTISRLEPEDFAVYYCQQY                 VLGQGTKVEIK(1)
>p30   GTLSLSPGERATLSCRASQSVSSSYLAWYQQKPGQAPRLLIYGASSRATGIPDRFSGSGSGTDFTLTISRLEPEDFAVYYCQQYGSSP            RTFGQGTKVEIK(1)
>p31   GTLSLSPGERATLSCRASQSVSSSYLAWYQQKPGQAPRLLIYGASSRATGIPDRFSGSGSGTDFTLTISRLEPEDFAVYYCQQYGSS            YTFGQGTKLEIK(2)
>p32   GTLSLSPGERATLSCRASQSVSSSYLAWYQQKPGQAPRLLIYGASSRATGIPDRFSGSGSGTDFTLTISRLEPEDFAVYYCQQYGS    PKS          GGTKVEIK(4)
>p33   GTLSLSPGERATLSCRASQSVSSSYLAWYQQKPGQAPRLLIYGASSRATGIPDRFSGSGSGTDFTLTISRLEPEDFAVYYCQQYGSS   N          FGGGTKVEIK(4)
>p34   GTLSLSPGERATLSCRASQSVSSSYLAWYQQKPGQAPRLLIYGASSRATGIPDRFSGSGSGTDFTLTISRLEPEDFAVYYCQQYGSS   R         TFGQGTKVEIK(1)
>p35   GTLSLSPGERATLSCRASQSVSSSYLAWYQQKPGQAPRLLIYGASSRATGIPDRFSGSGSGTDFTLTISRLEPEDFAVYYCQQYGSS   L         TFGGGTKVEIK(4)
>p36   GTLSLSPGERATLSCRASQSVSSSYLAWYQQKPGQAPRLLIYGASSRATGIPDRFSGSGSGTDFTLTISRLEPEDFAVYYCQQYGSS   QGKAE      FGQGTKVEIK(1)
>p37   GTLSLSPGERATLSCRASQSVSSSYLAWYQQKPGQAPRLLIYGASSRATGIPDRFSGSGSGTDFTLTISRLEPEDFAVYYCQQYGSSP              FGQGTKVEIK(1)
>p38   GTLSLSPGERATLSCRASQSVSSSYLAWYQQKPGQAPRLLIYGASSRATGIPDRFSGSGSGTDFTLTISRLEPEDFAVYYCQQYGSS              FGGGTKVEIK(4)
>p39   GTLSLSPGERATLSCRASQSVSSSYLAWYQQKPGQAPRLLIYGASSRATGIPDRFSGSGSGTDFTLTISRLEPEDFAVYYCQQYGSSP  P        WTFGQGTKVEIK(1)
>p40   GTLSLSPGERATLSCRASQSVSSSYLAWYQQKPGQAPRLLIYGASSRATGIPDRFSGSGSGTDFTLTISRLEPEDFAVYYCQQYGSSP              FGGGTKVEIK(4)
>p41   GTLSLSPGERATLSCRASQSVSSSYLAWYQQKPGQAPRLLIYGASSRATGIPDRFSGSGSGTDFTLTISRLEPEDFAVYYCQQYGSS   ESKE     YTFGQGTKLEIK(2)
>p42   GTLSLSPGERATLSCRASQSVSSSYLAWYQQKPGQAPRLLIYGASSRATGIPDRFSGSGSGTDFTLTISRLEPEDFAVYYCQQYGSSP  PLWLF      FGQGTKLEIK(2)
>p43   GTLSLSPGERATLSCRASQSVSSSYLAWYQQKPGQAPRLLIYGASSRATGIPDRFSGSGSGTDFTLTISRLEPEDFAVYYCQQYGSSP  N        YTFGQGTKLEIK(2)

VK1-17:    IQMTQSPSSLSASVGDRVTITCRASQGIRNDLGWYQQKPGKAPKRLIYAASSLQSGVPSRFSGSGSGTEFTLTISSLQPEDFATYYCLQHNSYP N             Jk
>p1    ARCDIQMTQSPSSLSASVGDRVTITCRASQGIRNDLGWYQQKPGKAPKRLIYAASSLQSGVPSRFSGSGSGTEFTLTISSLQPEDFATYYCLQHNSY  E         YTFGQGTKLEIK(2)
>p2    ARCDIQMTQSPSSLSASVGDRVTITCRASQGIRNDLGWYQQKPGKAPKRLIYAASSLQSGVPSRFSGSGSGTEFTLTISSLQPEDFATYYCLQHNSYP RAAGFP    FTFGPGTKVDIK(3)
>p3    ARCDIQMTQSPSSLSASVGDRVTITCRASQGIRNDLGWYQQKPGKAPKRLIYAASSLQSGVPSRFSGSGSGTEFTLTISSLQPEDFATYYCLQHNSY  TW         TFGQGTKVEIK(1)
>p4    ARCDIQMTQSPSSLSASVGDRVTITCRASQGIRNDLGWYQQKPGKAPKRLIYAASSLQSGVPSRFSGSGSGTEFTLTISSLQPEDFATYYCLQHNSYP VIDGE     YTFGQGTKLEIK(2)
>p5    ARCDIQMTQSPSSLSASVGDRVTITCRASQGIRNDLGWYQQKPGKAPKRLIYAASSLQSGVPSRFSGSGSGTEFTLTISSLQPEDFATYYCLQHNSYP HKSQ      LTFGGGTKVEIK(4)
>p6    ARCDIQMTQSPSSLSASVGDRVTITCRASQGIRNDLGWYQQKPGKAPKRLIYAASSLQSGVPSRFSGSGSGTEFTLTISSLQPEDFATYYCLQHNS    D            FGPGTKVDIK(3)
>p7    ARCDIQMTQSPSSLSASVGDRVTITCRASQGIRNDLGWYQQKPGKAPKRLIYAASSLQSGVPSRFSGSGSGTEFTLTISSLQPEDFATYYCL         PRPAPM    WTFGQGTKVEIK(1)
>p8    ARCDIQMTQSPSSLSASVGDRVTITCRASQGIRNDLGWYQQKPGKAPKRLIYAASSLQSGVPSRFSGSGSGTEFTLTISSLQPEDFATYYCLQH       TVRG      WTFGQGTKVEIK(1)
>p9    ARCDIQMTQSPSSLSASVGDRVTITCRASQGIRNDLGWYQQKPGKAPKRLIYAASSLQSGVPSRFSGSGSGTEFTLTISSLQPEDFATYYCLQHNSYP RGFA            PGTKVDIK(3)
>p10   ARCDIQMTQSPSSLSASVGDRVTITCRASQGIRNDLGWYQQKPGKAPKRLIYAASSLQSGVPSRFSGSGSGTEFTLTISSLQPEDFATYYCLQHNSYP I             FGPGTKVDIK(3)
>p11   ARCDIQMTQSPSSLSASVGDRVTITCRASQGVRNDLGWYQQKPGKAPKRLIYAASGLQSGVPSRFSGSGSGTEFTLTISSLQPEDFATYYCLQHNSYP PPQP      ITFGQGTRLEIK(5)
>p13   ARCDIQMTQSPSSLSASVGDRVTITCRASQGIRNDLGWYQQKPGKAPKRLIYAASSLQSGVPSRFSGSGSGTEFTLTISSLQPEDFATYYCLQHNSYP R         YTFGQGTKLEIK(2)
>p14   ARCDIQMTQSPSSLSASVGDRVTITCRASQGIRNDLGWYQQKPGKAPKRLIYAASSLQSGVPSRFSGSGSGTEFTLTISSLQPEDFATYYCLQHNSYP PSPRPL     TFGPGTKVDIK(3)
>p15   ARCDIQMTQSPSSLSASVGDRVTITCRASQGIRNDLGWYQQKPGKAPKRLIYAASSLQSGVPSRFSGSGSGTEFTLTISSLQPEDFATYYCLQHNS    VDPF         FGPGTKVDIK(3)
>p16   ARCDIQMTQSPSSLSASVGDRVTITCRASQGIRNDLGWYQQKPGKAPKRLIYAASSLQSGVPSRFSGSGSGTEFTLTISSLQPEDFATYYCLQHNSYP PS        YTFGQGTKLEIK(2)
>p17   ARCDIQMTQSPSSLSASVGDRVTITCRASQGIRNDLGWYQQKPGKAPKRLIYAASSLQSGVPSRFSGSGSGTEFTLTISSLQPEDFATYYCLQHNSY                GPGTKVDIK(3)
>p18   ARCDIQMTQSPSSLSASVGDRVTITCRASQGIRNDLGWYQQKPGKAPKRLIYAASSLQSGVPSRFSGSGSGTEFTLTISSLQPEDFATYYCLQHNSYP PTR        TFGQGTKVEIK(1)
>p19   ARCDIQMTQSPSSLSASVGDRVTITCRASQGIRNDLGWYQQKPGKAPKRLIYAASSLQSGVPSRFSGSGSGTEFTLTISSLQPEDFATYYCLQHNSYP QLM       YTFGQGTKLEIK(2)
>p20   ARCDIQMTQSPSSLSASVGDRVTITCRASQGIRNDLGWYQQKPGKAPKRLIYAASSLQSGVPSRFSGSGSGTEFTLTISSLQPEDFATYYC      RSIIVTPGRS YTFGQGTKLEIK(2)
>p21   ARCDIQMTQSPSSLSASVGDRVTITCRASQGIRNDLGWYQQKPGKAPKRLIYAASSLQSGVPSRFSGSGSGTEFTLTISSLQPEDFATYYCLQHNSYP PS         TFGQGTKLEIK(2)
>p22   ARCDIQMTQSPSSLSASVGDRVTITCRASQGIRNDLGWYQQKPGKAPKRLIYAASSLQSGVPSRFSGSGSGTEFTLTISSLQPEDFATYYCLQHNSYP PGVL       TFGQGTKVEIK(1)
>p23   ARCDIQMTQSPSSLSASVGDRVTITCRASQGIRNDLGWYQQKPGKAPKRLIYAASGLQSGVPSRFSGSGSGTEFTLTISSLQPEDFATYYCLQHNSYP PPQP      ITFGQGTRLEIK(5)
>p24   ARCDIQMTQSPSSLSASVGDRVTITCRASQGIRNDLGWYQQKPGKAPKRLIYAASSLQSGVPSRFSGSGSGTEFTLTISSLQPEDFATYYCLQHNSYP RDP       YTFGQGTKLEIK(2)
>p25   ARCDIQMTQSPSSLSASVGDRVTITCRASQGIRNDLGWYQQKPGKAPKRLIYAASSLQSGVPSRFSGSGSGTEFTLTISSLQPEDFATYYCLQHNSYP RAAGLP    FTFGPGTKVDIK(3)
>p26   ARCDIQMTQSPSSLSASVGDRVTITCRASQGIRNDLGWYQQKPGKAPKRLIYAASSLQSGVPSRFSGSGSGTEFTLTISSLQPEDFATYYCLQHNSYP LEP       YTFGQGTKLEIK(2)
>p27   ARCDIQMTQSPSSLSASVGDRVTITCRASQGIRNDLGWYQQKPGKAPKRLIYAASSLQSGVPSRFSGSGSGTEFTLTISSLQPEDFATYYCLQHNSYP RP        YTFGQGTKLEIK(2)
>p29   ARCDIQMTQSPSSLSASVGDRVTITCRASQGIRNDLGWYQQKPGKAPKRLIYAASSLQSGVPSRFSGSGSGTEFTLTISSLQPEDFATYYCLQHNSY  RGIHHL     TFGQGTKLEIK(2)
>p30   ARCDIQMTQSPSSLSASVGDRVTITCRASQGIRNDLGWYQQKPGKAPKRLIYGASSLQSGVPSRFSGSGSGTEFTLTISSLQPEDFATYYCLQHNSYP LEP       YTFGQGTKLEIK(2)
>p31   ARCDIQMTQSPSSLSAFVGDRVTITCRASQGIRNDLGWYQQKPGKAPKRLIYAASSLQSGVPSRFSGSGSGTEFTLTISSLQPEDFATYYCLQHNSY  E         YTFGQGTKLEIK(2)
>p32   ARCDIQMTQSPSSLSASVGDRVTITCRASQGIRNDLGWYQQKPGKAPKRLIYAASSLQSGVPSRFSGSGSGTEFTLTISSLQPEDFATYYCLQHNSY  LWSPL      TFGQGTKVEIK(1)
>p33   ARCDIQMTQSPSSLSASVGDRVTITCRASQGIRNDLGWYQQKPGKAPKRLIYAASSLQSGVPSRFSGSGSGTEFTLTISSLQPEDFATYYCLQHNSYP PCIRLM    YTFGQGTKLEIK(2)
>p34   ARCDIQMTQSPSSLSASVGDRVTITCRASQGIRNDLAWYQQSPGKAPKRLIYAASSLQSGVPSRFSGSGSGTEFTLTISSLQPEDFATYYCLQHNSY  E         YTFGQGTKLEIK(2)
>p36   ARCDIQMTQSPSSLSASVGDRVTITCRASQGIRNDLGWYQQKPGKAPKRLIYAASSLQSGVPSRFSGSGSGTEFTLTISSLQPEDFATYYCLQHNSYP PL        YTFGQGTKLEIK(2)
>p37   ARCDIQMTQSPSSLSASVGDRVTITCRASQGIRNDLGWYQQKPGKAPKRLIYAASSLQSGVPSRFSGSGSGTEFTLTISSLQPEDFATYYCLQHNSYP PD         TFGQGTKLEIK(2)
>p38   ARCDIQMTQSPSSLSASVGDRVTITCRASQGIRNDLGWYQQKPGKAPKRLIYAASSLQSGVPSRFSGSGSGTEFTLTISSLQPEDFATYYCLQHNSYP RL           FGPGTKVDIK(3)
>p39   ARCDIQMTQSPSSLSASVGDRVTITCRASQGIRNDLGWYQQKPGKAPKRLIYAASSLQSGVPSRFSGSGSGTEFTLTISSLQPEDFATYYCLQHNSYP           LTFGGGTKVEIK(4)
>p40   ARCDIQMTQSPSSLSASVGDRVTITCRASQGIRNDLGWYQQKPGKAPKRLIYAASSLQSGVPSRFSGSGSGTEFTLTISSLQPEDFATYYCLQHNSY  PEW        TFGQGTKVEIK(1)
>p41   ARCDIQMTQSPSSLSASVGDRVTITCRASQGIRNDLAWYQQSPGKAPKRLIYAASSLQSGVPSRFSGSGSGTEFTLTISSLQPEDFATYYCLQHNSYP RAAGFP    FTFGPGTKVDIK(3)
>p42   ARCDIQMTQSPSSLSASVGDRVTITCRASQGVRNDLGWYQQKPGKAPKRLIYAASGLQSGVPSRFSGSGSGTEFTLTISSLQPEDFATYYCLQHNSY  EY         TFGQGTKLEIK(2)
>p43   ARCDIQMTQSPSSLSASVGDRVTITCRASQGIRNDLGWYQQKPGKAPKRLIYAASSLQSGVPSRFSGSGSGTEFTLTISSLQPEDFATYYCLQHNSYP PES       YTFGQGTKLEIK(2)
>p45   ARCDIQMTQSPSSLSASVGDRVTITCRASQGIRNDLGWYQQKPGKAPKRLIYAASSLQSGVPSRFSGSGSGTEFTLTISSLQPEDFATYYCLQHN      SYMAK     YTFGQGTKLEIK(2)
>p47   ARCDIQMTQSPSSLSASVGDRVTITCRASQGIRNDLGWYQQKPGKAPKRLIYAASSLQSGVPSRFSGSGSGTEFTLTISSLQPEDFATYYCLQHNS     SRGPW      TFGQGTKVEIK(1)
>p48   ARCDIQMTQSPSSLSASVGDRVTITCRASQGIRNDLGWYQQKPGKAPKRLIYAASSLQSGVPSRFSGSGSGTEFTLTISSLQPEDVATYYCLQHNSYP HKSQ      LTFGGGTKVEIK(4)
```

Supplementary Fig. 1 I V(D)J sequencing. **a**, Graphical representation of a total of 3.95 million read pairs obtained from V(D)J sequencing displayed for each PCR product. Read pairs represent the number of paired end reads obtained from the sequencer. After merging (merged reads), the reads were selected based on the presence of the forward and reverse primer sequences which represent all rearrangements. The number of unique amino acid sequences after translating are depicted as unique rearrangements. These sequences were further filtered for the presence of the primer including 5 expected adjacent amino acids in the correct reading frame and the absence of stop codons to obtain potentially functional rearrangements. The isotype of the potentially functional rearrangements was determined by the presence of the protein tag SSKSTSGG (Cγ1) or CSRSTSES (Cγ4). In total, Cγ4 switch variants are very rare, summing up to an average of 0,76% of all functional transgenic VH gene rearrangements found. **b,** Selection of the most prominent sequences of potentially functional rearrangements of the transgenic human Ig genes found in the transgenic minipigs are shown as amino acid sequences. Rare Cγ4 switch variants are also included. The sequence of the transgenic V genes is depicted (in blue) above rearranged sequences, indicating the position of the amino acids corresponding to N(D)N, J and C regions. Where identified, D gene segments are underlined and named right to the sequence. The numbers in parenthesis in VH and Vk sequences indicate the corresponding J element used. Amino acid variants resulting from point mutations are shown in red. The Cγ4 region of switch variants is in brown colour. Same V gene sequences of Cγ4 switch variants are also often associated with Cγ1.

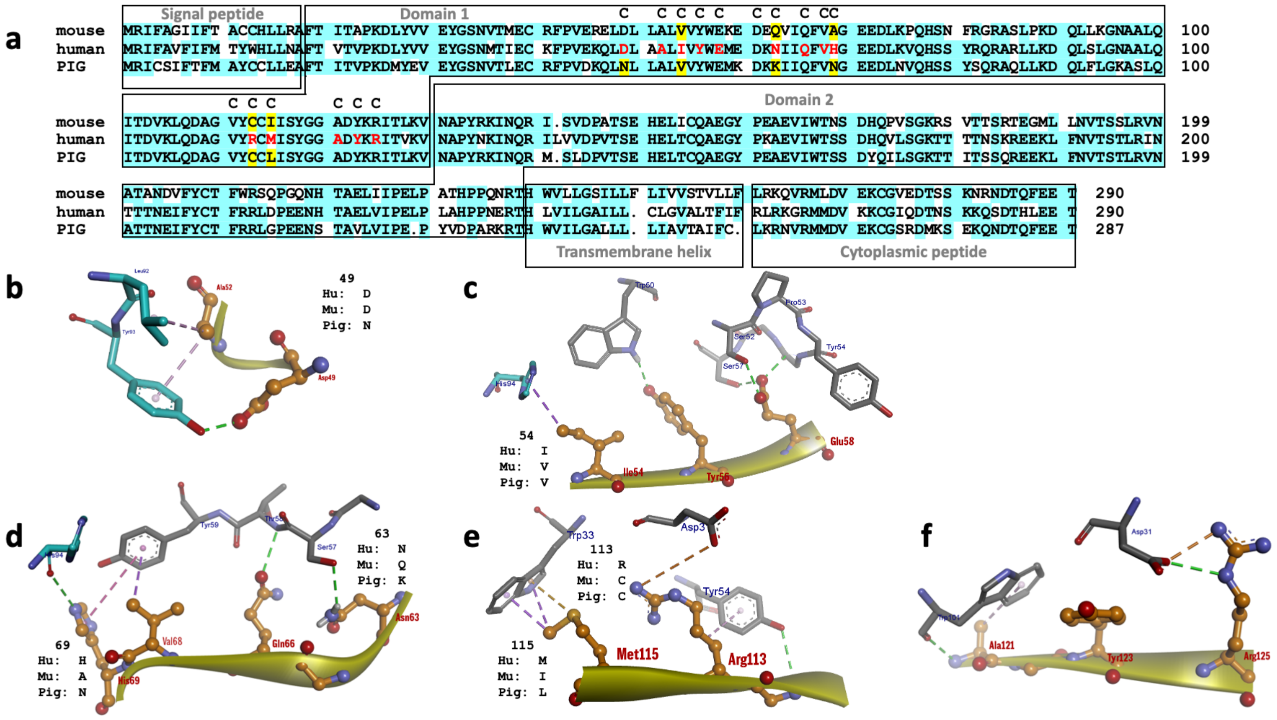

Supplementary Fig. 2 l Sequence and structural mapping of the different interactions of PD-L1 epitope amino acid side-chains with atezolizumab paratope residues. **a,** Sequence alignment of PD-L1 orthologs, human, mouse, and pig. Epitope residues are marked with a C (contact residues); an amino acid 1-letter code is marked in red when the contact is occurring exclusively via the side-chain atoms (amino acids with backbone contact only are kept in black). **b-f,** Molecular models of PD-L1 interaction with atezolizumab. Fourteen interacting side-chains show their contact with paratope residues. The corresponding residue position of PD-L1 interacting with atezolizumab is depicted for human (Hu) mouse (Mu) and pig (Pig) amino acids. Pig PD-L1 residues are indicated in the ball and stick mode (orange carbon atoms) and atezolizumab residues are represented in sticks (grey carbon atoms for the heavy variable region and cyan carbon atoms for the light variable region). PD-L1 and atezolizumab VH and VL are numbered consecutively starting from the first N-terminal residue.

Supplementary Table. 1 | List of Ab compounds.

Compound tested	Specificity	Clinical immunogenicity	Immunogenicity in huIgG1 tg mouse model*	Cross-reactivity mouse / pig
bevacizumab (Avastin)	VEGF	0,63 %	0/10 (8/10)	no[41] / yes[42]
daratumumab (Darzalex)	CD38	0 %	0/10 (4/10)	no / no
atezolizumab (Tecentriq)	PD-L1	30 %	10/10 (10/10)	yes / yes
cergutuzumab amunaleukin	CEA / IL2R	79 %	7/10 (7/10)[20]	yes (IL2R) / yes (IL2R)

*Given as the number of ADA-positive mice of 10 mice immunised in each group. Corresponding numbers for wild type mice are in parenthesis. Unpublished data, manuscript in preparation.

Supplementary Table. 2 | List of primers.

PCR amplification target	Primer name	Sequence 5'->3'
IGH-γ1-γ4 (transgene presence)	IGH_F<br>IGH_R	CTGTCCTACAGTCCTCAGGAC<br>GTGGCACTCATTTACCCGGAG
IGK (transgene presence)	IGK_F<br>IGK_R	TTGTGTGCCTGCTGAAT<br>GCCGCGTGTGAGTAGGTATA
BS (transgene presence)	BS_F<br>BS_R	ATGGCCAAGCCTTTGTCTC<br>GGCGAAGAACTCCAGCATGA
IGH-γ1-γ4 (ddPCR)	dIGH_F<br>dIGH_R	CACCCAGTGCGAGACGAC<br>CTCCTCCTCCCTCGCTCT
IGK (ddPCR)	dIGK_F<br>dIGK_R	GCTATGTAGAAGAGGCAGCT<br>ACCACCTTTCCACCATTACAA
GAPDH (ddPCR)	ddGAPDH_F<br>ddGAPDH_R	CTCAACGACCACTTCGTCAA<br>CCCTGTTGCTGTAGCCAAAT
First VH PCR (RNA sequencing)	Vh1-69_L<br>Vh4-59_L<br>Vh3-30_L<br>Vh3-23_L<br>Vh1-18_L<br>Cg1_4-rev_long	TGGCAGCAGCTACAGGTGTC<br>GCTCCCAGATGGGTCCTGT<br>CGTTGCTCTTTTAAGAGGTGTCC<br>GCTATTTTAAAAGGTGTCCAGTGTG<br>CAGCAACAGGTGCCCACT<br>GGTGTTGCTGGGCTTGTG
Nested VH CR (RNA sequencing)	VH1-18_A<br>VH1-69_A<br>VH3-23/30_A<br>VH4-59_A<br>Cg1_4-PCR_A	ATGGTATCAGCTGGGTGCGAC<br>AGGCACCTTCAGCAGCTATGC<br>GCAGACTCCGTGAAGGGCCG<br>AAGGGACTGGAGTGGATTGGG<br>AGTAGTCCTTGACCAGGCAGC
VK PCR (RNA sequencing)	Vk1-17_A<br>Vk3-20_A<br>Ck-PCR_A	GCCAGGTGTGACATCCAGATG<br>AGGCACCCTGTCTTTGTCTCC<br>CAGATGGTGCAGCCACAGTTC
