# Supplementary material for: A humanized minipig model for the toxicological testing of therapeutic recombinant antibodies
Source: Nat Biomed Eng. 2022 Sep 22;6(11):1248–56. doi: 10.1038/s41551-022-00921-2 (PMC9652145; doi:10.1038/s41551-022-00921-2)
Supplement: Supplementary file 2 — Reporting Summary [file 41551_2022_921_MOESM2_ESM.pdf]

nature portfolio

Corresponding author(s): Angelika Schnieke, Felix Weber

Last updated by author(s): Jun 27, 2022

Reporting Summary

Nature Portfolio wishes to improve the reproducibility of the work that we publish. This form provides structure for consistency and transparency in reporting. For further information on Nature Portfolio policies, see our Editorial Policies and the Editorial Policy Checklist.

Statistics

For all statistical analyses, confirm that the following items are present in the figure legend, table legend, main text, or Methods section.

n/a	Confirmed	
☐	☒	The exact sample size (*n*) for each experimental group/condition, given as a discrete number and unit of measurement
☐	☒	A statement on whether measurements were taken from distinct samples or whether the same sample was measured repeatedly
☐	☒	The statistical test(s) used AND whether they are one- or two-sided<br>*Only common tests should be described solely by name; describe more complex techniques in the Methods section.*
☒	☐	A description of all covariates tested
☒	☐	A description of any assumptions or corrections, such as tests of normality and adjustment for multiple comparisons
☐	☒	A full description of the statistical parameters including central tendency (e.g. means) or other basic estimates (e.g. regression coefficient) AND variation (e.g. standard deviation) or associated estimates of uncertainty (e.g. confidence intervals)
☒	☐	For null hypothesis testing, the test statistic (e.g. *F*, *t*, *r*) with confidence intervals, effect sizes, degrees of freedom and *P* value noted<br>*Give P values as exact values whenever suitable.*
☒	☐	For Bayesian analysis, information on the choice of priors and Markov chain Monte Carlo settings
☒	☐	For hierarchical and complex designs, identification of the appropriate level for tests and full reporting of outcomes
☒	☐	Estimates of effect sizes (e.g. Cohen's *d*, Pearson's *r*), indicating how they were calculated

Our web collection on statistics for biologists contains articles on many of the points above.

Software and code

Policy information about availability of computer code

Data collection	QX200 Droplet Generator and Reader were used for absolute quantification of target DNA. Optical density was measured at 405 nm using the VersaMax ELISA plate reader. The RNA integrity number (RIN) was determined by the Agilent 2100 Bioanalyzer. llumina sequencing libraries were prepared using Illumina TruSeq Nano DNA Sample Preparation Protocol. The sample libraries were sequenced on an Illumina MiSeq run using Paired End Sequencing for 2x300 cycles.
Data analysis	PCR-positive and PCR-negative droplets were counted to provide absolute quantification of target DNA in digital form using QuantaSoftTM software. OD values of standard curves were interpolated to calculate the concentration of human IgG1 in the serum. For the ADA IgG titres calculation, a cut-off value was determined for each study based on the mean OD at dilution 1:50 of all naïve samples multiplied with their 6-fold standard deviation. Baseline outliers were determined as beyond 1.5 times the interquartile range (IQR) of quartile 3, and were excluded. OD values of titrated serum samples were fitted using the 5th degree polynomial and titers were determined by the intersection of this curve with the determined threshold.<br><br>Both mates of overlapping paired end reads were merged using usearch tool version 0.667_i86linux32 and parameters -fastq_pctid 75 and -fastq_maxdiffs 25. Subsequently, reads were translated into amino acid sequences in the anticipated frame and filtered for the presence of 5 expected adjacent amino acids and the absence of stop codons to obtain potentially functional rearrangements.<br><br>Statistical analyses were performed by ordinary one-way analysis of variance (ANOVA) and Holm-Sidak's multiple comparisons test.<br>Data were analysed using GraphPad Prism 8.

For manuscripts utilizing custom algorithms or software that are central to the research but not yet described in published literature, software must be made available to editors and reviewers. We strongly encourage code deposition in a community repository (e.g. GitHub). See the Nature Portfolio guidelines for submitting code & software for further information.

Data

Policy information about availability of data

All manuscripts must include a data availability statement. This statement should provide the following information, where applicable:

- Accession codes, unique identifiers, or web links for publicly available datasets
- A description of any restrictions on data availability
- For clinical datasets or third party data, please ensure that the statement adheres to our policy

The data supporting the results in this study are available within the paper and its Supplementary Information. Source data for the figures are provided with this paper. The raw and analysed datasets generated during the study are available from the corresponding authors on request.

Field-specific reporting

Please select the one below that is the best fit for your research. If you are not sure, read the appropriate sections before making your selection.

☒ Life sciences ☐ Behavioural & social sciences ☐ Ecological, evolutionary & environmental sciences

For a reference copy of the document with all sections, see nature.com/documents/nr-reporting-summary-flat.pdf

Life sciences study design

All studies must disclose on these points even when the disclosure is negative.

Sample size	We did not conduct a formal power analysis to determine the sample size. We aimed to collect as many samples as was reasonably possible given the experimental constrains.
Data exclusions	No data were excluded from the experiments.
Replication	Reproducibility was tested by injecting four different monoclonal antibodies into wild-type and transgenic Göttingen mini-pigs. All ELISA measurements were performed in triplicates.
Randomization	The same type of material and animals were used for all experiments. Groups were formed based on the wild-type and transgenic animals.
Blinding	The veterinary who performed s.c. injections and collected the blood samples was blind to the statistical process.

Reporting for specific materials, systems and methods

We require information from authors about some types of materials, experimental systems and methods used in many studies. Here, indicate whether each material, system or method listed is relevant to your study. If you are not sure if a list item applies to your research, read the appropriate section before selecting a response.

Materials & experimental systems

n/a	Involved in the study	
☐	☒	Antibodies
☒	☐	Eukaryotic cell lines
☒	☐	Palaeontology and archaeology
☐	☒	Animals and other organisms
☒	☐	Human research participants
☒	☐	Clinical data
☒	☐	Dual use research of concern

Methods

n/a	Involved in the study	
☒	☐	ChIP-seq
☒	☐	Flow cytometry
☒	☐	MRI-based neuroimaging

Antibodies

Antibodies used	Mouse anti-Human IgG1 Fc Secondary Antibody, Biotin (Invitrogen #10467318), Biotin Mouse Anti-Human Ig κ Light Chain (BD Biosciences #555790), AffiniPure Goat Anti-Human IgG (H+L) (Jackson ImmunoResearch #109-005-003), Recombinant Anti-Kappa light chain antibody (Abcam ab124727), Rabbit anti huIgG H+L (OriGene #R1364HRP), HRP anti-human IgG (clone G18-145, Thermofisher #15838438), AffiniPure goat anti-swine IgG (H+L) (Jackson ImmunoResearch 114-035-003), Goat F(ab')2 anti-human κ light chain (Sigma-Aldrich, #SAB3701289), Mouse anti-human IgG heavy chain, HRP (BD #555788), Ig, κ Light Chain, mouse anti-human (BD #565232), Mouse anti-pig IgG (ProSci #1607).
Validation	Validation of each antibody was done under standard information offered by the supplier.

Animals and other organisms

Policy information about studies involving animals; ARRIVE guidelines recommended for reporting animal research

Laboratory animals	Adult Göttingen Minipigs of both sexes
Wild animals	The study did not involve wild animals.
Field-collected samples	The study did not involve samples collected from the field.
Ethics oversight	Government of Upper Bavaria, Germany (ROB-55.2-1-54-2532-6-13). The experiments were performed according to the German Welfare Act and the European Union Normative for Care and Use of Experimental Animals.

Note that full information on the approval of the study protocol must also be provided in the manuscript.
