# Supplementary figures and images for: A humanized minipig model for the toxicological testing of therapeutic recombinant antibodies
Source: Nat Biomed Eng. 2022 Sep 22;6(11):1248–56. doi: 10.1038/s41551-022-00921-2 (PMC9652145; doi:10.1038/s41551-022-00921-2)

**Figure 2a Uncropped Blots**

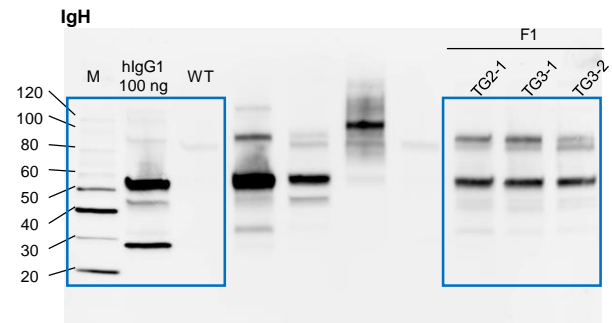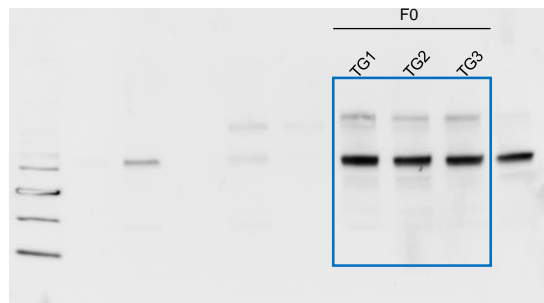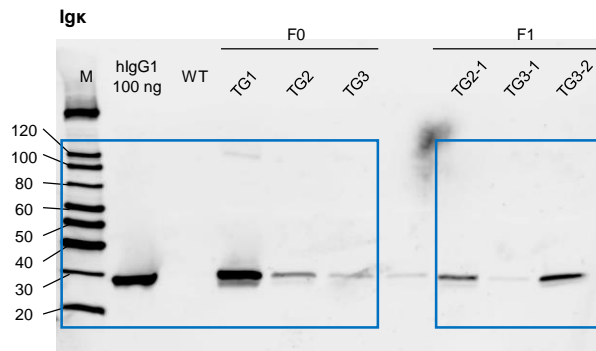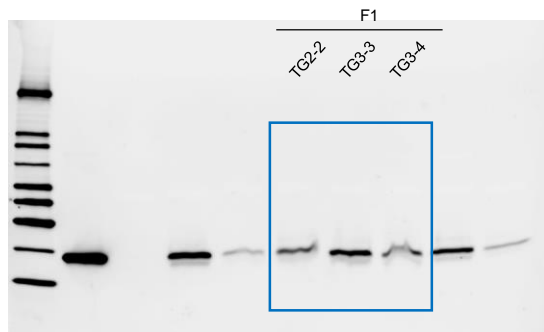

Supplement: Source data for Fig. 2 — Uncropped western blots for Fig. 2a. [file 41551_2022_921_MOESM4_ESM.pdf]
